# Characterization of ADME Gene Variation in Colombian Population by Exome Sequencing

**DOI:** 10.3389/fphar.2022.931531

**Published:** 2022-06-30

**Authors:** Daniel Felipe Silgado-Guzmán, Mariana Angulo-Aguado, Adrien Morel, María José Niño-Orrego, Daniel-Armando Ruiz-Torres, Nora Constanza Contreras Bravo, Carlos Martin Restrepo, Oscar Ortega-Recalde, Dora Janeth Fonseca-Mendoza

**Affiliations:** ^1^ Department of Molecular Diagnosis, Genética Molecular de Colombia SAS, Bogotá, Colombia; ^2^ Center for Research in Genetics and Genomics—CIGGUR, GENIUROS Research Group, School of Medicine and Health Sciences, Universidad Del Rosario, Bogotá, Colombia

**Keywords:** ADME, rare variants, exome sequencing, Latin America, pharmacogenomics, drug response

## Abstract

In genes related to drug pharmacokinetics, molecular variations determine interindividual variability in the therapeutic efficacy and adverse drug reactions. The assessment of single-nucleotide variants (SNVs) is used with growing frequency in pharmacogenetic practice, and recently, high-throughput genomic analyses obtained through next-generation sequencing (NGS) have been recognized as powerful tools to identify common, rare and novel variants. These genetic profiles remain underexplored in Latin-American populations, including Colombia. In this study, we investigated the variability of 35 genes included in the ADME core panel (absorption, distribution, metabolism, and excretion) by whole-exome sequencing (WES) of 509 unrelated Colombian individuals with no previous reports of adverse drug reactions. Rare variants were filtered according to the minor allele frequencies (MAF) <1% and potential deleterious consequences. The functional impact of novel and rare missense variants was assessed using an optimized framework for pharmacogenetic variants. Bioinformatic analyses included the identification of clinically validated variants described in PharmGKB and ClinVar databases. Ancestry from WES data was inferred using the R package EthSEQ v2.1.4. Allelic frequencies were compared to other populations reported in the public gnomAD database. Our analysis revealed that rare missense pharmacogenetic variants were 2.1 times more frequent than common variants with 121 variants predicted as potentially deleterious. Rare loss of function (LoF) variants were identified in 65.7% of evaluated genes. Regarding variants with clinical pharmacogenetic effect, our study revealed 89 sequence variations in 28 genes represented by missense (62%), synonymous (22.5%), splice site (11.2%), and indels (3.4%). In this group, *ABCB1*, *ABCC2*, *CY2B6*, *CYP2D6*, *DPYD*, *NAT2*, *SLC22A1*, and *UGTB2B7*, are the most polymorphic genes. *NAT2, CYP2B6* and *DPYD* metabolizer phenotypes demonstrated the highest variability. Ancestry analysis indicated admixture in 73% of the population. Allelic frequencies exhibit significant differences with other Latin-American populations, highlighting the importance of pharmacogenomic studies in populations of different ethnicities. Altogether, our data revealed that rare variants are an important source of variability in pharmacogenes involved in the pharmacokinetics of drugs and likely account for the unexplained interindividual variability in drug response. These findings provide evidence of the utility of WES for pharmacogenomic testing and into clinical practice.

## Introduction

Interindividual variability of therapeutic efficacy is determined by physiological, pathological, environmental and genetic factors. These differences are clinically important due to their influence in the generation of adverse drug reactions (ADRs). ADRs are a significant cause of morbidity and death worldwide, impacting patients’ health and the global economy (Aagaard et al., 2012; Formica et al., 2018). It has been estimated that the heritability of drug response can reach up to 90%, suggesting an important genetic influence in the regulation of this response and principally attributed to pharmacokinetic variability (Matthaei et al., 2015). Characterization of polymorphisms in actionable pharmacogenetic genes and their clinical application is potentially useful in drug selection, dosage optimization and prevention of unnecessary adverse effects. Currently, the assessment of high frequency (>5%) single nucleotide variants (SNVs) in genetic panels is the most used methodology in pharmacogenetic practice (van der Lee et al., 2020). However, the development of new genomic analysis technologies such as next generation sequencing (NGS) has made it possible to investigate the impact of rare variants, which in turn may explain the fraction of heritable variability not attributed to SNVs (Ingelman-Sundberg et al., 2018).

Recent studies have shown that approximately 80% of patients carry variants in genes involved in the metabolism of commonly prescribed drugs (Rabbani et al., 2016; Ji et al., 2018; Russell and Schwarz, 2020; van der Lee et al., 2020). Importantly, proteins involved in absorption, distribution, metabolism, and excretion (ADME) determine the pharmacokinetic profile of drugs and their high variability impacts the safety and pharmacological tolerance in ethnically different individuals and populations. The main determinant of this heterogeneity is the allele frequency of polymorphisms in ADME genes (Hovelson et al., 2017; Rodrigues et al., 2019). Considering such impact, several pharmaco-ethnicity studies have been carried out in cohort of individuals with predominant European ancestry. Whereas useful for these populations, pharmacogenetic research in poorly studied populations such as Latin America, which makes up 8.4% of the world population, remains limited (Rodrigues-Soares et al., 2020). Some genetic and population-based characteristics in pharmacogenes have recently been described in Latin America, with very low representation of Colombian population (Suarez-Kurtz and Parra, 2018; Rodrigues et al., 2019; Rodrigues-Soares et al., 2020). Given the presence of high interpopulation variability and the impact of the genetic background in the drug response, it is important to establish genomic profiles and large-scale DNA analysis in specific population, such as Colombia. In order to identify genetic variants potentially related to the safety and efficacy of common prescribed drugs, whole-exome sequencing (WES) was performed in individual with no previous reports of adverse drug reactions individuals from the Colombian population. This approach was followed by an innovative downstream bioinformatic analysis, which allowed the identification of common and rare variants in 35 genes described in the ADME core (including *VKORC1* and *DRD2* pharmacodynamic genes). For the analysis of rare variants, an optimized prediction framework was implemented to determine the functional impact of pharmacogenetic variants (Zhou et al., 2019). To the best of our knowledge, this is the first study performed in Colombian population that considers the use of WES to investigate ADME genes.

## Materials and Methods

### Samples and Data

Human subjects were recruited from a private laboratory: Genética Molecular de Colombia Ltda, in Bogotá, Colombia during 2017–2021. All individuals signed an informed consent form for the storage and molecular analysis of their samples. A total of 509 subjects were recruited for WES analysis for clinical purposes. All subjects had Colombian nationality, were not consanguineous and had not reports of previous adverse drug reactions (ADRs). Considering that this is the first study in ADME genes analyzed by NGS in Colombia, the sample size was calculated using previous reports generated by our group. The sample proportion (p) of 10.2% was established according to *CYP2C19* allelic frequencies in Colombian populations (Angulo-Aguado et al., 2021). Next the OpenEpi^1^ web-tool was utilized using the formula *n* = Nz2*p(1-p)/(α2(N-1)+z2*p(1-p), where the confidence level was set as 95% (*α* = 0.05, z = 1.96), the margin of error (e) was 3%, and the population size N = 8,000,000 for a finite population. The sample size was equal to 423, considering the potential loss of information 509 subjects were included in the study. This study was approved by the Ethics Committee of Universidad del Rosario (DV0005 1403-CV1281) and followed Helsinki declaration principles.

### Whole-Exome Sequencing

All samples were sequenced using Illumina technology. Exome library preparation and sequencing was performed by either Novogene (Beijing, China), using the Agilent SureSelect V6 kit (Agilent, Santa Clara, CA) and the NovaSeq 6000 equipment (Illumina Inc., San Diego, CA) (*n* = 167); or Theragen Etex Bio Institute (Suwon, Korea), using the Agilent SureSelect V4 kit (Agilent, Santa Clara, CA) and the HiSeq 4000 equipment (Illumina Inc., San Diego, CA) (*n* = 110); or Sophia Genetics (Athenas, Greece), using the clinical exome solution v.2 (CES2) and NextSeq 500 (Illumina Inc., San Diego, CA) (*n* = 232).

Patient’s DNA was obtained using the Quick-DNA 96 Plus kit (Zymo Research). Three micrograms of DNA per sample were verified for both quality and quantity and used for analysis Genomic DNA was randomly fragmented, and adapters were ligated to both ends of the fragments, with proper adenylation of DNA fragment 3ʹ ends. After PCR reaction, the biotinylated libraries were captured with streptavidin-coated magnetic beads. Index-labeled PCR amplicons were purified using the AMPure XP system and quantified using the Agilent Bioanalyzer 2100 system. Library quality was checked prior to sequencing and sequenced using a 150 bp paired-end approach. Raw data was obtained in FASTQ format, trimmed, and filtered to remove adapter sequences and low-quality reads. Quality control methodology included 1) discard a read pair if either read contains adapter contamination, 2) discard a read pair if more than 10% of bases are uncertain in either read and, 3) discard a read pair if the proportion of low-quality bases is over 50% in either read. >80% of total bases called had a Phred-scaled quality score greater than 30 (>Q30).

Filter reads were mapped to the reference genome hg19 (GRCh37) using the Burrows-Wheeler Aligner (BWA). Variant calling was done using GATK v3.8. A minimum of 6.5 Gb raw data was obtained and the percentage of reads properly mapped was >93.46% (21,492,427-122,546,997) per sample. Average mapping efficiency was >99%, with sequencing depth on target and coverage of target region >90x and >99.4%, respectively. Average fraction of target covered with >20x was >95%. Coverage uniformity (10x) was ≥90% and the average fraction of target covered with at least 10x, 20x, 50x and 100x was >98%, >90%, >52% and >30%, respectively. The average number of paired-end reads that mapped to the reference genome was 99,872,528 (99.86%). Library preparation and sequencing was performed by Novogene (Beijing, China), Theragen Etex Bio Institute (Suwon, Korea) and Sophia Genetics (Athenas, Greece) platforms. Variant call format (VCF) files were analyzed using the software Varseq (Golden Helix), which incorporated the following database annotations: Ensembl^2^, RefSeq^3^ (NCBI), ClinVar^4^, PharmaGKB^5^, dbSNFP Functional predictions, dbscSNV Splice altering predictions and gnomAD v2.1^6^. After variant quality filtering a total of 7,514,627 variants were obtained. The analysis included the ADME genes involved in absorption, distribution, metabolism and elimination processes of common drugs used in the clinic and described in the PharmaADME database^7^ (Table 1).

**TABLE 1:** ADME core gene panel.

Gen	Transcript (NCBI)	Class
*ABCB1*	NM_001348946.2	Transporter
*ABCC2*	NM_000392.5	Transporter
*ABCG2*	NM_000392.5	Transporter
*CYP1A1*	NM_001319217.2	Phase I
*CYP1A2*	NM_000761.5	Phase I
*CYP2A6*	NM_000762.6	Phase I
*CYP2B6*	NM_000767.5	Phase I
*CYP2C19*	NM_000769.4	Phase I
*CYP2C8*	NM_000770.3	Phase I
*CYP2C9*	NM_000771.4	Phase I
*CYP2D6*	NM_000106.6	Phase I
*CYP2E1*	NM_000773.4	Phase I
*CYP3A4*	NM_017460.6	Phase I
*CYP3A5*	NM_000777.5	Phase I
*DPYD*	NM_000110.4	Phase I
*DRD2*	NM_000795.4	Pharmacodynamic
*GSTM1*	NM_000561.4	Phase II
*GSTP1*	NM_000852.4	Phase II
*GSTT1*	NM_000853.3	Phase II
*NAT1*	NM_000662.8	Phase II
*NAT2*	NM_000015.3	Phase II
*SLC15A2*	NM_021082.4	Transporter
*SLC22A1*	NM_003057.3	Transporter
*SLC22A2*	NM_003058.4	Transporter
*SLC22A6*	NM_153276.3	Transporter
*SLCO1B1*	NM_006446.5	Transporter
*SLCO1B3*	NM_019844.4	Transporter
*SULT1A1*	NM_001055.3	Phase II
*TPMT*	NM_000367.4	Phase II
*UGT1A1*	NM_000463.3	Phase II
*UGT1A9*	NM_019076.4	Phase II
*UGT2B15*	NM_001076.4	Phase II
*UGT2B17*	NM_001077.3	Phase II
*UGT2B7*	NM_001074.4	Phase II
*VKORC1*	NM_024006.6	Pharmacodynamic

### Bioinformatic Analysis

Variants were stratified into two groups: Group 1. Molecular variants with known clinical significance, described in the public databases pharmGKB (clinical evidence level <4) and ClinVar; Group 2. Rare (MAF <1%) and novel molecular variants without clinical pharmacogenetic effect reported. For downstream analysis, all variants included in the group 1 were considered. For group 2, only molecular variants with potential pathogenicity were selected, these included LoF variants (nonsense, frameshift and splicing), and missense variants with functional impact according to a prediction framework optimized for pharmacogenetic genes (Zhou et al., 2019). Briefly, this framework uses multiple prediction metrics to improve the assessment of pharmacogenetic variants. First, individual scores for LRT, MutationAssessor, PROVEAN, VEST3 and CADD algorithms were computed using ANNOVAR software. Next, individual scores were classified as deleterious or neutral based on an ADME-optimized threshold value for each algorithm and assigned as 1 (deleterious) or 0 (functionally neutral). Thresholds were set according to those developed by Zhou et al. (2019) to assess the impact of pharmacogenetic variants. Finally, a composite score was obtained averaging optimized individual predictions (0 or 1). The optimized prediction value was compared with a conventional approach using default thresholds by VarSeq Sorting intolerant from tolerant (SIFT), Polymorphisms phenotyping 2 (PolyPhen2), MutationTaster, MutationAssessor and functional analysis through hidden Markov models (FATHMM). For splice site variants Adaptive boosting (ADA)/Random Forest (RF) scores (cutoff ≥0.6) and human splicing Finder (HSF) v3.1 were used to predict potential splicing alterations.

### Identification of Metabolizer Phenotypes

The metabolizing phenotypes were determined for the *ABCG2*, *CYP2B6*, *CYP2C9*, *CYP2C19*, *CYP3A5*, *DPYD*, *NAT2*, *SLCO1B1* and *TPMT*, according to the diplotype-phenotype information available in the PharmGKB clinical guidelines. Initially, the alleles were established based on SNVs information or previously described haplotypes. Given that our methodology did not allow us to identify copy number variants (CNVs) or structural variants for *CYP2D6* and these account for a large proportion of the genetic variability, CYP2D6 metabolizer phenotypes were not assessed. When it was not possible to define the allele because more than one variant was identified and these could be in cis or trans, all the potential diplotypes were considered and assigned to their corresponding metabolizing phenotypes. For the genes *CYP2C9, CYP2C19, CYP3A5, DPYD* and *TPMT*, the metabolizer phenotypes were classified as normal, intermediate and poor. For the genes *ABCG2* and *SLCO1B1*, the phenotypes were classified normal, decreased, and poor metabolizers. For *NAT2* the phenotypes were rapid, intermediate, intermediate/slow, slow and for *CYP2B6*, the phenotypes were normal, intermediate, rapid, ultra-rapid and poor metabolizers.

### Population Genetics Analysis

Population genetic data allelic frequencies were obtained from VarSeq Software. HWE deviation was estimated using Chi-square goodness-of-fit test with 1° of freedom. Supplementary Tables S1–S3 present all molecular variants identified (validated variants described in PharmGKB and ClinVar, and rare and/or novel MAF<1%). Latin American or general allelic frequencies were obtained from gnomAD v2.1 public database and compared with our data using Chi-square test and Bonferroni correction (Karczewski et al., 2020). For this analysis we considered only variants with clinical pharmacogenomic effect, this is PharmGKB level between 1A and 3 annotations and validated in ClinVar. Statistical significance was concluded at *p* < 0.05 (Supplementary Table S1). Ancestry analysis was conducted using the R package EthSEQ v2.1.4. We used the “SS2.Major” pre-computed reference model to infer ethnicity for each individual. This model was built using 1051 individuals from European (EUR), African (AFR), South Asian (SAS) and East Asian (EAS) populations obtained from the 1,000 Genome Project and considering 123,291 SNPs (Romanel et al., 2017).

## Results

### Demographic Characteristics

Characteristics of subjects are summarized in Table 2. Among the 509 Colombian individuals enrolled in this study, demographic characteristics were obtained from 476 of them. 49% of the population were male (*n* = 235) and 51% were female (*n* = 241). According to the Colombian regions classification described previously (Paredes et al., 2003), our sample was mainly from the Andean region (86%, *n* = 409), a population with predominant native ancestry (52%), and in minor proportion European and African influence, 45% and 3%, respectively (Salzano and Sans, 2014).

**TABLE 2:** Demographic characteristics.

	n	%
Sex		
Female	241	51
Male	235	49
Geographic region		
Andean	409	86
Caribbean	48	10
Orinoquian	10	2
Amazonian	9	2
Pacific	0	0

### Profiling of Molecular ADME Variants With Known Clinical Pharmacogenomic Effect

In the sample population, 89 known sequence variants were identified in 28 genes (Supplementary Table S1). These variants have been validated by pharmacogenetic relevant studies and included in PharmGKB or ClinVar with an evidence level of 1, 2, or 3. The most frequent pharmacogenetic known variants were missense (*n* = 55, 62%) followed by synonyms (*n* = 20, 22.5%), splicing (*n* = 10, 11.2%), indel (*n* = 3, 3.4%) and 3′ UTR (*n* = 1, 1.1%). Nonsense mutations were not found in this group of analysis. The highest number of variants identified per gene was 8 (*ABCB1*), while other genes had no polymorphisms (*GSTM1, GSST1, NAT1, SLC22A6, UGT1A1, UGT1A9* and *UGT2B17*). Highly polymorphic genes included *ABCB1, DPYD, CY2B6, CYP2D6, NAT2, SLC22A1, ABCC2*, and *UGTB2B7*, with between 5 and 8 SNPs per gene (Table 3). Regarding metabolism phases, 39 variants were identified in 12 phase I genes, 28 variants in 8 transport genes, and 17 variants in 6 phase II (Supplementary Table S1). The identified molecular variants exhibited a wide range of allelic frequencies with values between 0.098% and 89%. Most variants (*n* = 53, 59.5%) were classified as frequent (MAF >5%), whereas 13.5 (*n* = 12) had allele frequencies between 1% and 5% and 13.5% (*n* = 12) corresponded to rare variants (MAF <1%). In the rare variants group, *DPYD* and *CYP2D6* genes exhibited the highest number of variants, 11 and 10, respectively (Table 3). Remarkably, 89% of the sampled population has at least one pharmacogenetically informative variant (Supplementary Table S1).

**TABLE 3:** Total variants indentified per gen.

Category	Gen	PharmGKB and clinvar clinical pharmacogenetic effect variants	Rare missense and LOF variants	Total variants
Transporter	*ABCB1*	8	9	17
Transporter	*ABCC2*	5	9	14
Transporter	*ABCG2*	1	3	4
Phase I	*CYP1A1*	1	6	7
Phase I	*CYP1A2*	1	5	6
Phase I	*CYP2A6*	1	0	1
Phase I	*CYP2B6*	7	5	12
Phase I	*CYP2C19*	2	7	9
Phase I	*CYP2C8*	3	2	5
Phase I	*CYP2C9*	4	3	7
Phase I	*CYP2D6*	6	10	16
Phase I	*CYP2E1*	1	3	4
Phase I	*CYP3A4*	2	4	6
Phase I	*CYP3A5*	3	6	9
Phase I	*DPYD*	8	11	19
Others	*DRD2*	2	1	3
Phase II	*GSTP1*	2	3	5
Phase II	*GSTT1*	0	1	1
Phase II	*NAT1*	0	4	4
Phase II	*NAT2*	6	1	7
Transporter	*SLC15A2*	1	2	3
Transporter	*SLC22A1*	6	8	14
Transporter	*SLC22A2*	1	4	5
Transporter	*SLC22A6*	0	7	7
Transporter	*SLCO1B1*	4	5	9
Transporter	*SLCO1B3*	2	6	8
Transporter	*SLCO1B7*	0	4	4
Phase II	*SULT1A1*	1	5	6
Phase II	*SYCE1*	0	1	1
Phase II	*TPMT*	2	1	3
Phase II	*UGT1A1*	0	3	3
Phase II	*UGT2B15*	1	4	5
Phase II	*UGT2B17*	0	4	4
Phase II	*UGT2B7*	5	4	9
Pharmacodynamic	*VKORC1*	3	1	4
	Total	89	152	241

LOF, loss of function.

**FIGURE 1:**
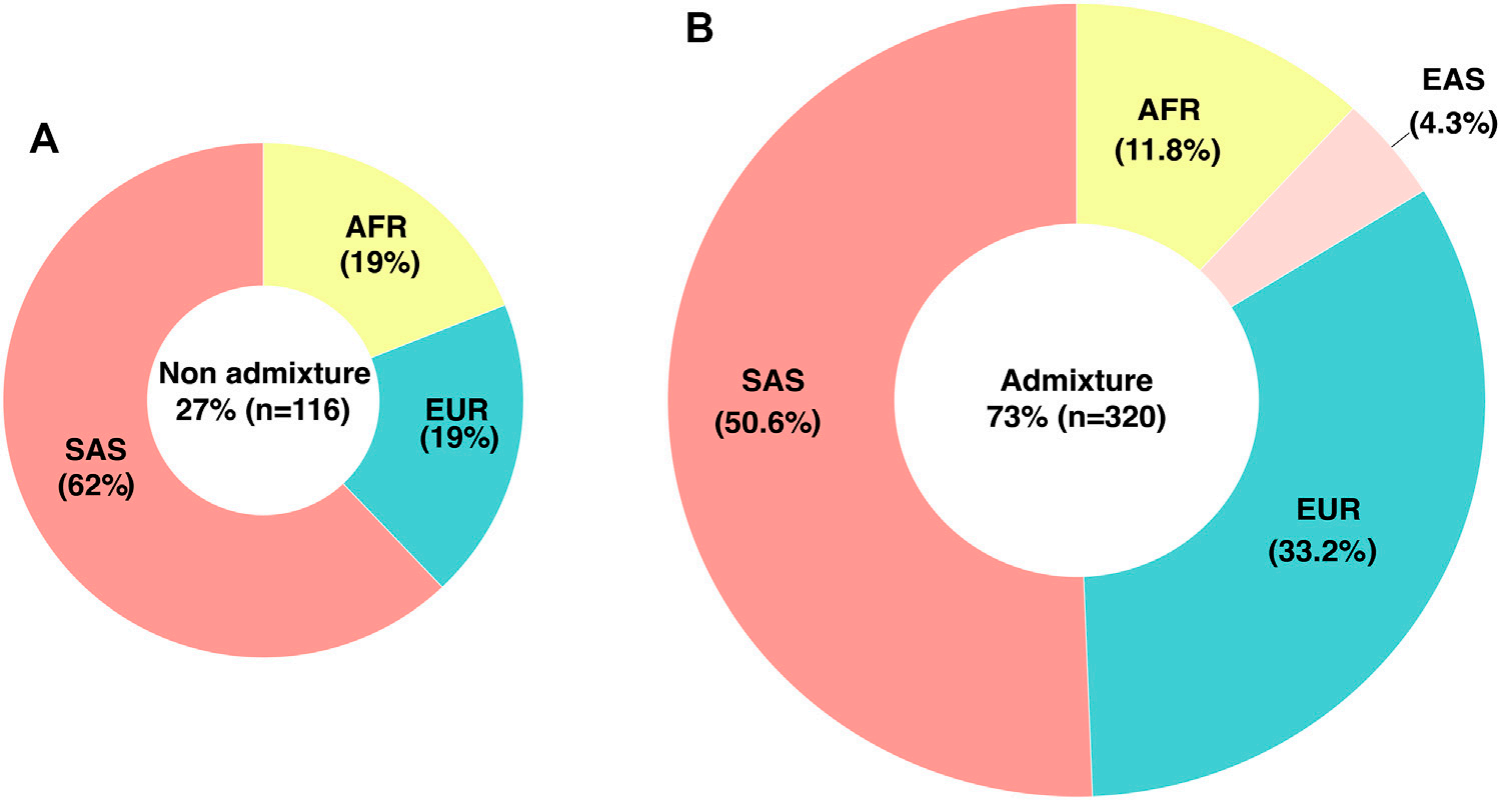
Genetic ancestry and admixture in the studied population determined by WES. **(A)** Percentage and distribution of individuals classified as non-admixed **(B)** Percentage and ancestry contribution of individuals classified as admixed. EUR, European; AFR, African; SAS, South Asian; EAS, East Asian.

**FIGURE 2:**
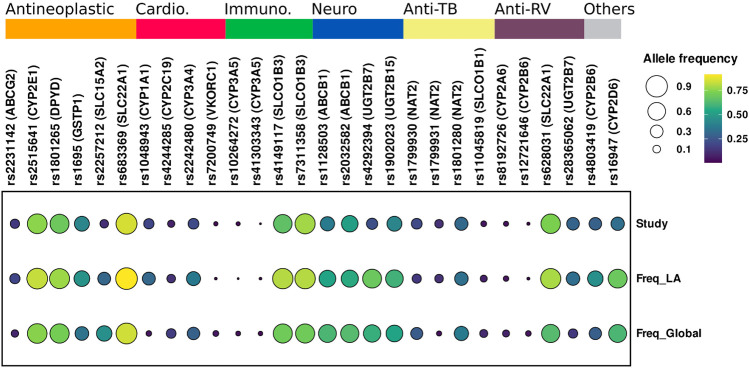
Comparison of allelic frequency of variants with clinical pharmacogenetic effect. Study, Data generate in this study; Freq_LA, Allele frequency Latin-American population; Freq-Global, Allele frequencies global. Genes and rs related to Antineoplastic, Cardio, Cardiovascular; Immuno, Immunosuppressant; Neuro, Neuropsychiatric; Anti-TB, Anti-tuberculosis; Anti-RV, Antiretrovirals drugs.

WES ancestry analysis using the R package EthSEQ was possible for 436 individuals (85.7%) and showed that 73% (*n* = 320) had admixed ancestry with a contribution of AFR 11.8%, EUR 33.2%, SAS 50.6%, and EAS 4.3% components. (Figure 1) (Supplementary Table S4). The comparison of the allelic frequencies for the variants in our population to those reported in Latin American population in the gnomAD public database indicated that 13 of them (rs1128503, rs4292394, rs16947, rs2257212, rs2242480, rs1902023, rs4803419, rs4149117, rs112843, rs2515641, rs683369, rs1801265 and rs10264272) presented significant differences (*p* < 0.05) (Table 4). Interestingly, 92% of the polymorphisms occurred at significantly lower frequencies in the sampled population, which is relevant due to their implication in the pharmacokinetics of commonly used drugs in the clinic, including methadone (rs4292394), tacrolimus (rs2242480), oxazepam (rs1902023), efavirenz (rs4803419), carboplatin (rs4149117), and cyclosporine (rs1128503) among others (Figure 2). Furthermore, relevant for the immunosuppressive response to tacrolimus, the *CYP3A5* c.624G > A (rs10264272) polymorphism allele frequency was 6.5 times higher in our sample than in general Latin American population (Table 4).

**TABLE 4:** Functionally relevant SNPs allelic frequencies comparison.

Gen	Rs	Drug	Allele frequencies^a^	gnomAD_general_allele frequencies	*p*_value	*p*_value_bonferroni correction	gnomAD_latin_allele frequencies	*p*_value	*p*_value_bonferroni correction
*DPYD*	rs1801265	Antineoplastic	0.698	0.720	0.141	1	0.776	<0.01	0.02^b^
*SLC22A1*	rs683369	Antineoplastic	0.854	0.846	0.499	1	0.914	<0.01	0.01^b^
*SLC15A2*	rs2257212	Antineoplastic	0.126	0.455	<0.01	<0.01^b^	0.309	<0.01	<0.01^b^
*CYP2E1*	rs2515641	Antineoplastic	0.755	0.749	0.663	1	0.832	<0.01	0.01^b^
*ABCG2*	rs2231142	Antineoplastic	0.154	0.094	<0.01	<0.01^b^	0.189	0.048	1
*CYP1A1*	rs1048943	Cardiovascular	0.190	0.050	<0.01	<0.01^b^	0.315	<0.01	<0.01^b^
*CYP3A4*	rs2242480	Cardiovascular	0.208	0.291	<0.01	<0.01^b^	0.370	<0.01	<0.01^b^
*CYP2C19*	rs4244285	Cardiovascular	0.087	0.167	<0.01	<0.01^b^	0.129	<0.01	0.50
*VKORC1*	rs7200749	Cardiovascular	0.028	0.055	<0.01	0.02^b^	0.009	<0.01	0.65
*CYP3A5*	rs10264272	Immunosuppressant	0.024	0.036	0.031	1	0.004	<0.01	0.04^b^
*CYP3A5*	rs41303343	Immunosuppressant	0.005	0.030	<0.01	<0.01^b^	0.009	0.242	1
*SLCO1B3*	rs7311358	Immunosuppressant	0.785	0.705	<0.01	<0.01^b^	0.809	0.208	1
*ABCB1*	rs1128503	Neuropsychiatric	0.382	0.614	<0.01	<0.01^b^	0.534	<0.01	<0.01^b^
*UGT2B7*	rs4292394	Neuropsychiatric	0.232	0.577	<0.01	<0.01^b^	0.691	<0.01	<0.01^b^
*UGT2B15*	rs1902023	Neuropsychiatric	0.421	0.537	<0.01	<0.01^b^	0.593	<0.01	<0.01^b^
*NAT2*	rs1799930	Anti-tuberculosis	0.209	0.270	<0.01	<0.01^b^	0.150	<0.01	0.14
*NAT2*	rs1801280	Anti-tuberculosis	0.322	0.385	<0.01	0.01^b^	0.342	0.365	1
*NAT2*	rs1799931	Anti-tuberculosis	0.130	0.041	<0.01	<0.01^b^	0.131	0.937	1
*SLCO1B1*	rs11045819	Anti-tuberculosis	0.053	0.111	<0.01	<0.01^b^	0.082	0.014	1
*CYP2A6*	rs8192726	Antiretrovirals	0.040	0.083	<0.01	<0.01^b^	0.047	0.467	1
*CYP2B6*	rs12721646	Antiretrovirals	0.014	0.050	<0.01	<0.01^b^	0.012	0.717	1
*UGT2B7*	rs28365062	Antiretrovirals	0.297	0.155	<0.01	<0.01^b^	0.342	0.040	1
*CYP2D6*	rs16947	Others	0.334	0.614	<0.01	<0.01^b^	0.688	<0.01	<0.01^b^
*CYP2B6*	rs4803419	Others	0.292	0.282	0.475	1	0.455	<0.01	<0.01^b^

a Present study.

b Statistically significant.

### Molecular Variants Without Clinical Pharmacogenetic Effects Reported

#### Novel and Rare Loss-of-Function Variants

From the 509 subjects included in the study, LoF and splicing variants with a MAF <1% were filtered and 31 variants in 23 pharmacogenes were found. From these, 13 encoded for phase I enzymes, 10 for phase II enzymes, and 8 for transporters. Genes encoding for phase I enzymes had more variants compared to the others (Supplementary Table S5). Concerning LoF variants, 24 variants were found, 13 of them are generated by nonsense mutations and 11 by indels. Most of the nonsense and indels variants were observed on phase I and II genes.

Additionally, 7 splicing variants with an allele frequency of less than 1% and with an ADA score ≥0.6 and RF score ≥0.6 were found in 6 genes (Supplementary Table S2). Most of the rare splicing variants were detected on transport genes.

One variant observed in the *SULT1A1* gene (c.138T > A; p.Tyr46Ter) was identified in 2 individuals in homozygous state. Another rare variant, *SLCO1B3* c.971-2A > G was found in one individual in homozygous state. For the *CYP3A4* and *CYP1A1* genes, 2 new variants were identified, c.1321_1322delAA (p. Asn441Leufs*28) and c.1043-1G > A, respectively.

#### Rare Missense Variants

A total of 334 missense variants with MAF<1%, corresponding to 50 novel variants were identified in the population. These variants were analyzed using a functional prediction framework optimized for pharmacogenetic assessments (OPF score). This model integrates individual assessments of five algorithms (LRT, MutationAssessor, PROVEAN, VEST3 and CADD) and optimized thresholds for ADME-genes, achieving 93% for sensitivity and specificity for the discrimination of deleterious and neutral pharmacogenetic variants. Individual scores for each algorithm and the composite score for the ADME-optimized model are shown in Supplementary Table S3. For the analysis, the threshold was set at ≥ 0.6 obtaining 121 variants (36.2%) potentially predicted as deleterious (Supplementary Table S3). The scores obtained using this approach were compared with a conventional model using the number of algorithms that agreed regarding potential functional effect and default thresholds. The conventional model identified 139 potential deleterious variants (threshold ≥3/6 algorithms) with 104 variants that were common for both approaches, corresponding to 74.8% of the ADME-optimized model. The ADME-optimized scores were highly correlated with the conventional score (*R* ^2^ = 0.76, *p* value < 0.01).

### Metabolizer Phenotypes in the Colombian Population

**FIGURE 3:**
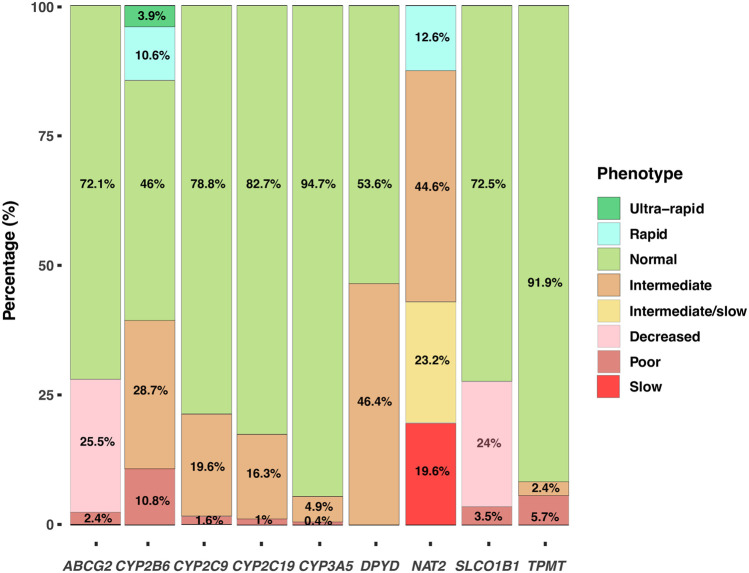
Metabolizer Phenotypes. Distribution of the metabolizer phenotypes based on the diplotypes for the genes *ABCG2*, *CYP2B6*, *CYP2C9*, *CYP2C19*, *CYP3A5*, *DPYD*, *NAT2*, *SLCO1B1* and *TPMT*.

With the results obtained by WES we identified the SNV and/or the haplotypes that defined the alleles for the genes *ABCG2*, *CYP2B6*, *CYP2C9*, *CYP2C19*, *CYP3A5*, *DPYD*, *NAT2*, *SLCO1B1* and *TPMT*. The diplotypes allowed us to assign the metabolizer phenotype for each induvial and determine its frequency (Figure 3). The diplotype-phenotype relationships for each gene were obtained from data available in the PharmGKB clinical guidelines. *NAT2* exhibited the highest variability, with only 12.6% of the population classified as rapid acetylator. On the other hand, a substantial proportion of the studied subjects were normal metabolizers for *CYP3A5* and *TPMT*, 94.7% and 91.9%, respectively. Interestingly, a high proportion of intermediate metabolizers was identified in *DPYD* and *NAT2* genes, 46.4% and 44.6%, respectively. Overall, the percentage of slow metabolizers was low, with values ranging between 0.4% and 5.7%, except for slow acetylators, which frequency was higher (19.6%) (Figure 3). Our results showed that a high proportion of the individuals analyzed in our study had at least one compromised metabolizer phenotype, which in turn may have an impact on the treatment response to several drugs commonly used in clinical practice.

## Discussion

The interindividual variation in therapeutic response to medications is partially attributed to pharmacokinetic differences related to polymorphisms in ADME genes (Klein et al., 2019). Despite the importance of such variants in pharmacogenomic medicine, studies in Latin American populations are limited. In this study, we aimed to characterize the pharmacogenetic variation in a sample of 509 Colombian individuals using a NGS approach.

Our investigation identified a total of 152 rare variants including: 1) A large proportion of missense variants with a potential functional effect according to a pharmacogenetic optimized algorithm (79.6%, *n* = 121), 2) LoF variants (20.4%, *n* = 31), and variants with clinical pharmacogenetic effect (7.9%, *n* = 12). These observations support the importance of a high-throughput approach as a useful strategy to detecting rare and novel variants potentially involved in clinical pharmacogenomics (Kozyra et al., 2017; Hocevar et al., 2019; Klein et al., 2019). Enrichment of pharmacogenetic variation by potentially pathogenic rare variants, mainly missense, has been observed in several studies worldwide, with low evolutionary constraints as a potential explanation for this phenomenon (Nelson et al., 2012; Tennessen et al., 2012; Genomes Project Consortium et al., 2015; Ingelman-Sundberg et al., 2018; Suarez-Kurtz and Parra, 2018). Importantly, the use of strategies based on microarrays to genotype common SNPs may be insufficient to detect these variants, highlighting the importance of more robust analyses to enhance the implementation of personalized medicine (Arbitrio et al., 2021).

We implemented an optimized score (OPF) described by Zhou et al. (2019) to assess the functional impact of pharmacogenetic missense variants. Although this approach revealed a high correlation with a conventional model (Adjusted *R* ^2^ = 0.76, *p* < 0.001), the optimized model identified variants as potentially pathogenic, missed by the conventional algorithms. Considering that some algorithms, conventionally used for the prediction of pathogenicity derive their result from the analysis of amino acid conservation between species (SIFT, FATHMM, FATHMM-2 and Mutation Assesor), the OPF score has the advantage of incorporating multiple criteria that exceed the power of discrimination of pathogenicity in pharmacogenetic variants. On the other hand, future research in pathogenicity prediction is required to improve such discrimination power, particularly taking into account the high number of variants in the population and the low evolutionary pressure on genes important in drug metabolism (Pshennikova et al., 2019; Zhou et al., 2019).

Our study found that cytochrome P450 (*CYP540*) genes harbor many rare variants in agreement with previous reports (Fujikura et al., 2015; Wright et al., 2018; Tasa et al., 2019; da Rocha et al., 2021). Considering that approximately 79% of widely used drugs are metabolized by enzymes of the *CYP450* family, the impact of rare variants potentially related to the generation of ADRs in the sampled population is significant (Phillips et al., 2001; Zanger and Schwab, 2013; Fujikura et al., 2015; Tornio and Backman, 2018). For this gene family, known pharmacogenetic variants accounted for just 4.6% of all variants, whereas variants with *in silico* pathogenicity prediction and/or functional impact were present in 33.6% of the population. The majority of rare *CYP450* variants were found in *CYP2D6* (6.2%) and remarkably some of these variants were considerably more frequent in our population (p.Tyr355Cys, MAF = 5.6%). This result illustrates the importance of characterizing proper pharmacogenomic profiles in specific Latin American populations in order to advance pharmacogenetic medicine (Naranjo et al., 2018).

Rare variants were also significantly represented in genes related to drug transport (37.5%, *n* = 57), which could potentially lead to pharmacokinetic disturbances, clinically evidenced by the development of drug resistance mediated by decreased uptake or increased efflux (Franke et al., 2010). New variants with potential pathogenic effect were found in 14.9% of the total population. Recently, Ingelman-Sundberg et al. (2018) carried out an NGS study in 60,706 individuals where they evaluated 208 ADME genes and identified that 83% of the variants had not been previously reported. This finding, consistent with our investigation, suggests that new variants may explain a considerable fraction of the pharmacogenetic variability in drug response.

Rare variants with potential or known clinical pharmacogenetic effects accounted for 68% (*n* = 164), whereas common variants accounted for 32% (*n* = 77), meaning that rare variants were 2.1 times more frequent than common variants in our studied population. Based on this approach, we estimate that 68% of functional variability in the core ADME genes can be attributed to rare variants. A previous report indicated that rare variants account for 30%–40% functional variability in pharmacogenes (Kozyra et al., 2017). Noteworthy, most drug responses are complex traits and there is growing evidence supporting the role of rare variants as important contributors to the unexplained differences in pharmacological responsiveness (Hocevar et al., 2019).

Despite the advantages of incorporating WES in pharmacogenomics, identification of variants in promoter and deep intronic regions is limited and should be considered. For instance, polymorphisms such as CYP2C19*17, *VKORC1 -1639 G > A* o *UGT1A1* (TA)n, related to the response to clopidogrel, warfarin and glucuronidation of many drugs, are not identified by WES (Russell et al., 2021). These actionable variants are responsible for adverse reactions to drugs in standard dosages and, therefore, their genotyping is recommended by The Clinical Pharmacogenetics Implementation Consortium (CPIC) (Johnson et al., 2011; Scott et al., 2011). Moreover, novel variants in non-exonic regions might have a functional impact in drug responses, for example, our group recently analyzed the promoter region of *CYP2C19* identifying novel SNVs that could potentially disrupt its binding activity to transcription factors and alter clopidogrel response (Angulo-Aguado et al., 2021). In addition, important pharmacogenetic variants such as CYP3A5*3, related to immunosuppressive drug response, or CYP2C19*35, related to clopidogrel response, are located in intronic regions not assessed by WES (Genvigir et al., 2020; Angulo-Aguado et al., 2021).

In addition to identifying new and rare variants, exome sequencing allows the identification and characterization of variants with known pharmacogenomic effect (Russell et al., 2021). Making use of information available in pharmacogenomic databases such as PharmGKB and ClinVar, we estimated that 80% of the evaluated genes carried at least one variant with a described clinical effect. By focusing on variants with PharmGKB clinical annotations between levels 1A to 3, it was possible to identify 86 molecular changes related to variant-drug combinations of clinical relevance (Whirl-Carrillo et al., 2021). Noteworthy, 71.4% of the genes in which molecular variants were identified belong to the VIP (very important pharmacogenes), group either due to their role in the metabolism of many drugs or because they contain variants that can potentially contribute to severe drug responses (www.pharmgkb.org).

The *ABCB1, ABCC2*, *CYP2B6, CYP2D6, DPYD, NAT2, SLC22A1* and *UGT2B7* exhibited the highest polymorphism level, with 5 to 8 variants per gene. The *CYP2D6* (cytochrome P450 2D6) and *DPYD* (dihydropyrimidine dehydrogenase) genes have the strongest evidence for translational applications with extensive information from clinical guidelines and drug label annotations for more than 50 widely used medications (Amstutz et al., 2018; Lunenburg et al., 2020). *CYP2D6* is one of the best-characterized phase I drug-metabolizing enzymes (Taylor et al., 2020). There are 125 clinical annotations and pharmacogenomic clinical guidelines for at least 48 *CYP2D6* substrate drugs, providing strong support for therapeutic decision-making genetic testing. In the sampled population, five variants generated alleles with reduced or null enzymatic activity (CYP2D6*10, CYP2D6*4, CYP2D6*6 y CYP2D6*17), with the highest allelic frequencies for *10 and *4 alleles, 10% and 13%, respectively. Despite that this allele definition is based on the core allele criteria developed by PharmVar and PharmGKB, defined as “only sequence variations that cause an amino acid change or impact function by changing expression levels or interfere with splicing,” the identification of alleles, haplotypes and diplotypes based on WES information represents a considerable challenge in pharmacogenetics (Nofziger et al., 2020). Consequently, some tools that use computational approximations that infer the most likely diplotype based on a known haplotype catalogue defined by PharmVar have been developed [e.g., Cypiripi (Numanagic et al., 2015), ASTROLABE (Twist et al., 2016), Aldy (Numanagic et al., 2018) and Stargazer (Numanagic et al., 2018)]. However, such algorithms have not been evaluated systematically and each of them has its own limitations. The presence of pseudogenes (e.g., *CYP2D7*), structural variants and CNVs, make difficult to determine the diplotypes, for that reason, alternative approaches such as third-generation sequencing have been proposed and constitute and active area of research (Chen et al., 2021).

The *ABCB1* gene is of particular interest in the study population due to the high frequency of molecular variants with clinical impact: 8 variants with allele frequencies up to 52%. The *ABCB1* gene encodes for P-glycoprotein, a protein associated with the metabolism of 56 drugs. The two SNPs with the highest frequency in the sampled population (rs2032582 and rs1045642) have been functionally validated, showing alterations at the protein level or in efflux capacity, and substantial impact on systemic drug exposure and toxicity (Franke et al., 2010). Overall, these results support the importance of single genetic variants in the response to multiple drugs, including codeine and tamoxifen for which there are more than 200 clinical annotations (Brousseau et al., 2007; Teh et al., 2012; Province et al., 2014). Many of the known variants identified in this study have extensive clinical annotations, highlighting the importance of characterizing the genetic diversity in ADME genes. This characterization is particularly important to advance pharmacogenomic medicine in neglected populations with high degree of ethnic diversity, such as Colombian and other Latin American populations (Rodrigues et al., 2019).

Interestingly we identified a significant contribution of Asian ancestry in our population. Previous studies using ancestry-informative markers (AIMs) in Colombian population have demonstrated that ancestry is variable between different geographical regions. For the Andean region, the geographical area where most of our samples come from, the contributions reported in these studies were AFR 7.4%, EUR 58%, and Native American 34.6% (Ossa et al., 2016). Differences in these percentages could be due to distinct methodological approaches. Importantly, our WES ancestry analysis revealed a complex admixture pattern which could explain the differences in pharmacogenetic variant frequencies when compared to other populations. These observations require further analysis and highlight the importance of including understudied populations in pharmacogenetic research.

The comparison of the allelic frequencies between the Colombian population and these of public databases showed significant differences (*p* < 0.05) for 39 variants when compared to global frequencies and 13 variants when compared to Latin American frequencies. Several interesting differences in allelic frequencies were observed in variants related to multiple medical areas, including oncology, cardiovascular medicine, neuropsychiatry, immunology, and infectious diseases (Supplementary Table S1 and Figure 2).

The sampled population has statistically significant lower allele frequencies in pharmacogenetic relevant variants such as CYP2C19*2, related with high platelet reactivity in clopidogrel treatment (Pereira et al., 2019); CYP2D6*4, related to poor metabolism that impacts in the pharmacokinetic properties of multiple drugs (Taylor et al., 2020), and DPYD*9 related to toxicity in oncologic treatment with fluorouracil (Zhang et al., 2007; Saif et al., 2016). Conversely, higher allele frequencies for other variants, renders the sampled population more susceptible to ADRs caused by rosuvastatin (rs2231142) (DeGorter et al., 2013; Lee et al., 2013), isoniazid (rs1799931) and fluorouracil (rs17376848) (Bose et al., 2011). Furthermore, the allele frequency in our population is 6 times higher for CYP3A5*6 compared to Latin American gnomAD data. This polymorphism has been related to variability in tacrolimus immunosuppressive response (Birdwell et al., 2015).

Considering the discussed findings, we developed two pharmacogenetic models based on frequently used drugs, 5-Fluorouracil (5-FU) and warfarin, incorporating molecular evidence and potential applications in the pharmacogenomic clinical setting. The antineoplastic drug 5-fluorouracil (5-FU) is an antimetabolite agent with wide usage for patients older than 60 years and to date remains a key component of several chemotherapy schemes for solid tumors (Kadoyama et al., 2012). 5-FU activity is regulated through a complex pharmacokinetic pathway, and variants in more than 20 genes have been related to this drug response (*DPYD*, *TYMS*, *MTHFR*, *ABCB1*, and *GSTP1*) (Muhale et al., 2011).

*DPYD* codifies the enzyme dihydropyrimidine dehydrogenase (DPD), responsible of more than 80% of 5-FU primary drug metabolism (Scartozzi et al., 2011). Our study identified eleven rare genetic variants in this gene. Given the increased risk of toxicity related to high 5-FU plasmatic levels in individuals carrying non-WT alleles, it is plausible that rare variants have a potential functional impact, as reported in previous studies, and are of clinical interest (Henricks et al., 2018). Interestingly, some polymorphisms identified in the Colombian population showed high allelic frequencies for genotypes related to 5-FU toxicity, for example the DPYD*9 (69.8%) and DPYD*5 (21.6%) alleles. Functional studies for these alleles indicate a significant reduction in the enzymatic activity potentially related to increased drug plasmatic concentration leading to toxicity (Kuilenburg et al., 2016). Moreover, the present study identified a total of 52 variants in other pharmacogenes related to 5-FU pharmacokinetics and individual response (*ABCB1, ABCG2, CYP2C9, CYP1A2, CYP2A6, CYP2C8, GSTP, GSTT1, TPMT, UGT1A1*). Among these variants, 44% (*n* = 23) represent missense variants, 42% (*n* = 22) correspond to known clinical pharmacogenetic effect variants, and 14% (*n* = 7) rare LoF variants. Previous studies have demonstrated that approximately 30% of 5-FU inter-individual cytotoxicity variation can be attributed to genetic factors, highlighting the importance of considering both common and rare genetic variants to explain the drug response variability (Weng et al., 2013). Pharmacogenomics and personalized medicine have an essential role in cancer therapy, where drug failure may result in tumor progression and death. Additionally, 5-FU ADRs such as neutropenia, pyrexia, pulmonary embolism, thrombocytopenia, and leukopenia have the potential to be life-threatening (Kadoyama et al., 2012). In accordance with these considerations, the Swiss Agency of Therapeutic Products (Swissmedic) issued a 5-FU label warning recommending *DPYD* genetic testing.

For our second pharmacogenetic model we analyzed warfarin, an oral anticoagulant broadly used to prevent and treat thromboembolic disease. This drug is a racemic mixture of R and S enantiomers, metabolized by *CYP1A1, CYP1A2, CYP2C8, CYP2C9, CYP2C19,* and *CYP3A4* (Rettie et al., 1992; Hirsh et al., 1998; Kim et al., 2012). In a previous study, we reported that *CYP2C9* and *VKORC1* genotypes accounted for 44.4% of the overall warfarin maintenance dose variability (Galvez et al., 2018). *CYP2C9* exhibited reduced genetic variability compared to global and Latin American populations, represented in the sampled population by the alleles most frequently associated with poor metabolism (CYP2C9*2 and CYP2C9*3). Remarkably, our study found 50 additional variants in warfarin pharmacogenes (*ABCB1, CYP1A1, CYP1A2, CYP2C8, CYP2C19,* and *CYP3A4*), of which 54% (*n* = 27) correspond to missense variants, 34% (*n* = 17) had known clinical pharmacogenetic effect, and 12% (*n* = 6) were rare LoF variants. Because warfarin has a narrow therapeutic window and pharmacogenetics is a crucial factor involved in interindividual variability to drug response, the study of common and rare variants constitutes a useful clinical strategy to guide and personalize drug selection and dosage. In agreement with these observations, warfarin pharmacogenomics studies have demonstrated to be cost-effective and increase the quality-adjusted life-years (QALYs) per patient (You, 2011).

Our work presents the first study that characterizes the pharmacogenomic landscape of ADME genes in a Colombian population using whole-exome sequencing data. This study suggests that rare variants may be a major contributor to the unexplained pharmacogenomic interindividual variability to drug response and illustrates the use of NGS analysis as a useful tool for therapeutic decision-making.

## Study Limitations

The present study has several limitations worth noting. First, exome sequencing is not able to identify variants in regulatory and deep intronic regions which might have important consequences in gene expression and activity. Second, our methodology did not allow us to identify copy number variants (CNVs) or structural variants for *CYP2D6* and therefore CYP2D6 metabolizer phenotypes were not assessed.

The functional impact of rare variants was evaluated by an *in-silico* approach. Whereas useful and increasingly reliable, more robust approximations such as *in vivo* assays might shed light into the functional role of these variants. Finally, despite all samples and variants meet the requirements of quality control, rare variants were not confirmed by Sanger sequencing, a technique considered the “gold standard” for genotype determination.

## Conclusion

Exome sequencing represents a powerful and useful tool to study pharmacogenetic variability in ADME genes. Our study involved a comprehensive analysis of rare and known clinical pharmacogenetic variants in Colombian population, demonstrating that rare variants represented an important fraction of the genetic variability in pharmacogenetic loci, potentially related to drugs pharmacokinetics modulation. Our results provide compelling evidence that such approach is useful for Latin American and other neglected populations in order to understand their pharmacogenomic profile and advance in the clinical implementation of pharmacogenomic medicine.

## Data Availability

The authors consider that the datasets presented in this article are not readily available because the nature of this research contains information that could compromise the participants’ privacy, they did not agree to share their data publicly. Requests to access the datasets should be directed to DF-M.

## Footnotes

1
http://www.openepi.com/Menu/OE_Menu.htm

2
https://www.ensembl.org/index.html

3
https://www.ncbi.nlm.nih.gov/refseq/

4
https://www.ncbi.nlm.nih.gov/refseq/

5
https://www.pharmgkb.org/

6
https://gnomad.broadinstitute.org/

7
http://www.pharmaadme.org/
